# College Dispensary

**Published:** 1847-02

**Authors:** 


ARTICLE XIV.

### COLLEGE DISPENSARY.

This noble charity, established by the Faculty of Rush Medical College, has been adopted as a county institution by the Commissioners of Cook county, and well may they be proud of an institution which so liberally scatters its blessings amongst the sick poor. There have been over two hundred prescriptions made for more than one hundred patients since the opening of the session of the College, in November.

In addition to the extensive relief afforded the poor sufferers, there has another most important end been attained by it. The prescriptions were made before the medical class—thus teaching the young men how to practice, and affording them an opportunity of witnessing the effects of treatment upon a large variety of diseases. After entering upon the duties of the profession, the students who have witnessed the treatment of these cases, meeting with the diseases, will recognize them as old acquaintances—enemies—with the weapons of warfare against whom they are already familiar, both as to their nature and application—and they go forward in practice with confidence and success.

The following diseases, as shown by the record of the Dispensary, many of which will form interesting reports for the Journal when drawn up by the clerk, were presented, and served as illustrations for clinical lectures on practice to the class:

Hypertrophy of the heart; dyspepsia; intermittent fever, with almost all its complications; enlargement of the spleen; conjunctivitis, acute and chronic; ascitis; anasarca; chronic pleuritis; neuralgia; dysmenorhœa; gastritis; peritonitis, chronic; hysteria; gastro enteric irritation; scabies; jaundice; bronchitis; erysipelas, influenza, &c.

And the following were presented illustrating the surgical clinic:—Enlarged tonsils; old laxation of elbow joint; indolent ulcers; abscesses; nasal polypi; caries of bones and polypus of the ear; strabismus; ulceration of stump of amputated leg; necrosis; hernia; fractures, of metacarpal bones and of neck of the femur; incised wound of knee; encysted tumor; morbus coxarius, with many others of less importance.

These required the following operations, which were successfully performed by Prof. Brainard before the class:—Amputations; removal of three carious metatarsal bones, done while the patient was under the influence of the sulphuric ether from inhaling its vapor, patient suffering but little pain; operation for strabismus; removal of tonsils; reduction of old laxation of elbow joint; removal of nasal polypi, of polypi from the ear; application of truss; extirpating tumors; operation for fistula in ano, &c. &c.

Ample trial was made of many of the new remedies and modes of treatment recommended by the profession. And it is a pleasing consideration, that while most of the patients have been discharged cured, not one has died. G.N.F.

